# Expanding Mauser Bullets

**Published:** 1900-06-23

**Authors:** 


### EXPANDING MAUSER BULLETS.

SIR WILLIAM STOKES,[1] writing on the subject of the Mauser bullet and the wounds produced by it, points out that, notwithstanding all that was said in the earlier stages of the war as to the Mauser being a merciful and humane weapon, later experience has gone to show that the injuries produced by it have been of a much more serious character than those at first described. He specially refers to certain remarks made by Sir William MacCormac, Mr. Treves, Mr. Dent, and others who advocate the desirability of "masterly inactivity" in this department of surgery, and goes on to say that his experience in the Maritzburg military hospitals and in the general hospital, Mooi River, compel him to hold opinions which differ largely from the views mentioned. He believes that the gunshot wounds met with in the earlier part of the campaign differed materially in character from those observed of late, and that the increasing gravity of the wounds has been the cause of the increased difficulty which has arisen in keeping them aseptic. This change he attributes to two causes. First, the frequent conversion by the Boers of the Mauser bullet into an expanding one, either by removing a small portion of the case from the apex of the bullet, thus converting it into a "soft-nosed" one, or by making longitudinal slits round the case, and he adds that when recently at Ladysmith he got ample evidence of the means which were adopted by the enemy to increase the gravity of the wounds they inflicted. Secondly, another cause for the greater severity of the wounds observed of late has been that the ranges have been much closer than they were formerly, it being clearly established that the closer the range the more serious is the injury likely to be. In illustration of his conclusions, he gives details of a series of cases in which such grave injuries were produced by these bullets as to necessitate amputation of limbs.

[1] Brit. Med. Jour., June 16.

